# Controllable Technology for Thermal Expansion Coefficient of Commercial Materials for Solid Oxide Electrolytic Cells

**DOI:** 10.3390/ma17051216

**Published:** 2024-03-06

**Authors:** Ya Sun, Dun Jin, Xi Zhang, Qing Shao, Chengzhi Guan, Ruizhu Li, Fupeng Cheng, Xiao Lin, Guoping Xiao, Jianqiang Wang

**Affiliations:** 1 Shanghai Institute of Applied Physics, Chinese Academy of Sciences, Shanghai 201800, Chinaliruizhu@sinap.ac.cn (R.L.); chengfupeng@sinap.ac.cn (F.C.); linxiao@sinap.ac.cn (X.L.); 2 China State Shipbuilding Corporation Seago System Technology Co., Ltd., Shanghai 200001, China; 3 School of Materials Science and Engineering, Jingdezhen Ceramic University, Jingdezhen 333403, China; shaoqing@sinap.ac.cn

**Keywords:** thermal expansion coefficient, solid oxide electrolytic cells, quenching

## Abstract

Solid oxide electrolysis cell (SOEC) industrialization has been developing for many years. Commercial materials such as 8 mol% Y_2_O_3_-stabilized zirconia (YSZ), Gd_0.1_Ce_0.9_O_1.95_ (GDC), La_0.6_Sr_0.4_Co_0.2_Fe_0.8_O_3−δ_ (LSCF), La_0.6_Sr_0.4_CoO_3−δ_ (LSC), etc., have been used for many years, but the problem of mismatched thermal expansion coefficients of various materials between cells has not been fundamentally solved, which affects the lifetime of SOECs and restricts their industry development. Currently, various solutions have been reported, such as element doping, manufacturing defects, and introducing negative thermal expansion coefficient materials. To promote the development of the SOEC industry, a direct treatment method for commercial materials—quenching and doping—is reported to achieve the controllable preparation of the thermal expansion coefficient of commercial materials. The quenching process only involves the micro-treatment of raw materials and does not have any negative impact on preparation processes such as powder slurry and sintering. It is a simple, low-cost, and universal research strategy to achieve the controllable preparation of the thermal expansion coefficient of the commercial material La_0.6_Sr_0.4_Co_0.2_Fe_0.8_O_3−δ_ (LSCF) through a quenching process by doping elements and increasing oxygen vacancies in the material. Commercial LSCF materials are heated to 800 °C in a muffle furnace, quickly removed, and cooled and quenched in 3.4 mol/L of prepared Y(NO_3_)_3_. The thermal expansion coefficient of the treated material can be reduced to 13.6 × 10^−6^ K^−1^, and the blank sample is 14.1 × 10^−6^ K^−1^. In the future, it may be possible to use the quenching process to select appropriate doping elements in order to achieve similar thermal expansion coefficients in SOECs.

## 1. Introduction

Energy development is related to people’s well-being. Hydrogen energy is regarded as the most promising clean energy in the 21st century, and many countries and regions around the world have conducted extensive research on hydrogen energy. The medium- and long-term plans for the development of the hydrogen energy industry (2021–2035) emphasize the need to promote research into solid oxide electrolysis cells (SOECs) as a hydrogen production technology to promote the high-quality development of the hydrogen energy industry, and to assist in achieving carbon neutrality and carbon peaking goals.

SOECs are high-efficiency electrochemical energy storage and conversion devices. These devices can convert H_2_O (g) and CO_2_ into usable fuels such as hydrogen and syngas when powered on. The development of SOECs not only reduces carbon dioxide emissions, but also promotes the recycling of resources [1]. SOECs are mainly composed of an anode functional layer, electrolytes, and a cathode functional layer [2,3,4,5]. 

Many reasons may cause the degradation of SOECs under high-temperature conditions for a long time [6]. Ghamarinia et al. [7] reviewed some degradation factors on SOEC performance. Non-destructive testing analysis of SOECs using electrochemical impedance spectroscopy technology can clearly determine the factors that lead to cell performance degradation and their impact on cell impedance growth [8,9], such as attenuation factors leading to increasing ohmic impedance, polarization impedance, or gas diffusion impedance [10]. 

To meet commercial usage requirements, SOEC methods need to satisfy a minimum of 5 years of long-term usage. The mismatch of thermal expansion coefficients between different components of cells can lead to degradation, delamination, or fracture, which is one of the obstacles to the commercialization process of SOECs. The most commonly used positive electrode material on the air side of SOECs is cobalt containing perovskite materials, such as Sm_0.5_Sr_0.5_CoO_3−δ_, La_0.6_Sr_0.4_Co_0.2_Fe_0.8_O_3−δ_ (LSCF), Ba_0.5_Sr_0.5_Co_0.8_ Fe_0.2_O_3−δ_ (BSCF), and SrNb_0.1_Co_0.9_O_3−δ_ (SNC). This is because they have excellent oxygen reduction activity and high conductivity. But their thermal expansion coefficients (TECs) are very high, which can be attributed to the thermal reduction of cobalt ions, related phase transitions, and thermal activation transitions of Co-d orbital electron spin states [11].

LSCF perovskite oxide has high electronic conductivity and oxygen ion conductivity, making it a promising commercial material. But the thermal expansion coefficient of this material is very high. It has been reported that the thermal expansion coefficient (TEC) of LSCF is 17.5 × 10^−6^ K^−1^ at 800 °C, which is 70% higher than the TEC of 8 mol% Y_2_O_3_-stabilized zirconia (YSZ) (10.5 × 10^−6^ K^−1^) and 40% higher than Gd_0.1_Ce_0.9_O_1.95_ (GDC) (12.5 × 10^−6^ K^−1^) [12]. The mismatch of thermal expansion between materials will make it difficult to achieve rapid start-up of the SOEC stack and make it unstable in a high-current operation. 

To solve the interface contact problem caused by the mismatched thermal expansion coefficients of cells, scholars have made a lot of efforts in the past [13,14,15,16,17,18] (such as element doping [19]) to introduce A-site defects into perovskite [20] and composites of perovskite electrode materials with electrolyte materials [21]. The tedious modification strategy forces scientists to try new materials [22]. However, these new materials are rare in practical commercial applications because the development of new materials and the final commercial batch preparation process require a large number of experiments for verification. New sintering processes for cells [23] are being explored in order to reduce the occurrence of micro gaps at the interface of cells due to the intolerable stress caused by the interface during high-temperature sintering. 

During 2021 and 2022, ‘*Nature*’ reports on the interface delamination problem of SOECs once again drew the attention of scientists to solving this problem. In 2022, a method was reported to improve material interface contact by increasing the roughness of the electrode surface with acid treatment [24]. This method has to some extent improved the stability performance of the cell, but the acid-treated material causes certain damage to the crystal structure of the material. At the same time, acid brushing on the sintered electrolyte indicates that the local reaction rate of the electrolyte is uncontrollable, which can easily cause electrolyte perforation and impact the existing cell preparation process. However, this method only increases the interface contact area, but does not fundamentally solve the problem of interface delamination caused by the mismatched thermal expansion coefficients of materials. In 2021, ‘*Nature*’ reported the introduction of a negative thermal expansion coefficient compensation strategy and the preparation of composite electrodes with matching thermal expansion coefficients through the method of “addition and subtraction” [11]. The thermal expansion compensation strategy is to react and sinter cobalt containing perovskite materials with a “negative thermal expansion” material, and offset the negative impact of the positive thermal expansion coefficient of the perovskite materials through the “thermal shrinkage and cold expansion” characteristics of the negative thermal expansion material, thereby forming a new composite material that matches the electrolyte thermal expansion coefficient [25,26]. This composite material exhibits excellent electrochemical performance when prepared as an electrode. However, the introduction of materials with negative thermal expansion coefficients can easily reduce the catalytic activity of raw materials and introduce unknown interfacial reactions. More unfortunately, this method is limited to specific materials and does not have universality, which makes it unable to be applied to commercial material batch-processing processes [27]. It is worth noting that the doping of the material Y element and the absence of the material Sr element in this work can effectively reduce the thermal expansion coefficient of the raw material. 

Based on the current strategy of solving the thermal expansion coefficient of materials that is not suitable for the industrial processing of materials, the quenching process is introduced into the research on material modifications of SOECs. Through the quenching process, the controllable preparation of the thermal expansion coefficient of the commercial material LSCF is achieved through element doping and increasing material oxygen vacancies by adjusting the type and concentration of the quenching solution. The quenching process was initially used in the field of iron making. It not only allows for the doping of material elements, but also for the preparation of more oxygen vacancy concentrations in materials [28]. The doping of material elements can significantly improve the conductivity and catalytic activity of materials [29,30,31]. Li et al. [32] prepared Y-doped SrTiO_3_ with a solid-state reaction method, and studied the effect of Y doping on the sintering performance and conductivity of Y_x_Sr_1−x_TiO_3_. When the yttrium content in Y_x_Sr_1−x_TiO_3_ is less than 0.09, Y doping can improve the conductivity of SrTiO_3_. Tahini et al. [33,34] showed that doping can facilitate the formation of oxygen vacancies in materials. Increasing the oxygen vacancy concentration of the material can not only reduce the thermal expansion coefficient of the material, but also improve its conductivity. Hu et al. [35] reported that the introduction of oxygen vacancies significantly reduces the orthogonal strain and thermal expansion anisotropy of MoO_3_, resulting in a decrease of about 30% in the thermal expansion coefficient of MoO_3_. In addition, its conductivity increases with the increase in oxygen vacancies in the material. Niu et al. [36]. designed a simple solution reduction method to modify the surface defects of perovskite materials and studied the relationship between oxygen vacancy concentration and electrochemical performance. Experimental and theoretical calculations showed that an appropriate oxygen vacancy concentration is beneficial for obtaining a large number of active sites, which can significantly improve the ORR activity of materials. 

More importantly, the quenching process meets the batch processing requirements of commercial materials. It only requires micro-treatment on the surface of the raw material, and does not have an impact on the mixing of cell paste, cell sintering, or other cell preparation processes. Therefore, it can be directly applied to commercial processes. Comparing the quenching process reported in the literature with the existing modified material thermal expansion coefficient method, it can clearly be seen that the quenching process method is simpler, more feasible, and has universality. The specific data are shown in Table 1.

## 2. Experimental Materials and Instruments

Y(NO_3_)_3_·6H_2_O was purchased from China National Pharmaceutical Group Chemical Reagent Co., Ltd., Shanghai, China. purity: 99.99%, company: WoKai, weight: 500 g, country: China, city: Shanghai. LSCF commercial materials were purchased from Shanghai Hydrogen Cheng Technology Co., Ltd. Shanghai, China., and the muffle furnace was purchased from Hefei Kejing. A transmission electron microscope (TEM), JEOL JEM-2100HR, Pleasanton, CA, USA) and X-ray diffraction (XRD) (Bruker, Mannheim, Germany, specification model D8 Advance) with Cu kα radiation at 2θ from 5° to 90° were used at a scan rate of 10°/min. The thermal expansion instrument adopts the model number as specified (manufacturer: NETZSCH, Selb, Germany, specification model: DIL 402C).

## 3. Material Thermal Expansion Coefficient Test

Heat the commercial LSCF material in a muffle furnace to 800 °C and then quickly remove, quench, and cool it in 3.4 mol/L of prepared Y(NO_3_)_3_. Soak it for 3 min using the vacuum filtration method, wash the processed LSCF material thoroughly and collect it, and place it in a 60 °C oven for backup. 

**Figure 1:**
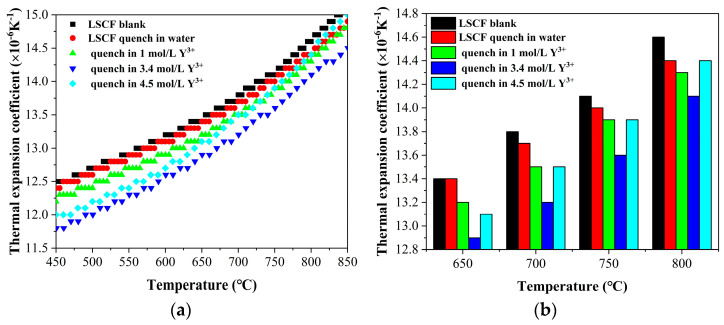
Thermal expansion coefficient test. (**a**) Scatter plot of thermal expansion coefficient with temperature variation (**b**) Histogram of thermal expansion coefficient with temperature variation.

When the quenched LSCF powder is dried and in the muffle furnace, raise the temperature from 20 °C to 1000 °C at a rate of 5 °C/min. Maintain a constant temperature for 2 h and sinter the powder into shape (sinter into a cylinder with a fixed diameter of 6 mm and a length of 10 mm using a special grinding tool). The thermal expansion coefficient of the material is measured using a thermal expansion instrument (DIL 402C) with a heating rate of 1 °C/min, rising from 20 °C to 800 °C, as shown in Figure 1. The quenching process can effectively reduce the thermal expansion coefficient of the LSCF material. Quenching treatment is effective in solutions with different Y^3+^ concentrations (1 mol/L Y^3+^ means LSCF quenched in 1 mol/L Y(NO_3_)_3_, others are also calculated accordingly). With an increasing concentration of quenching solution within a certain range, the modified LSCF has a smaller coefficient of thermal expansion. But when the concentration of the solution exceeds the range, the increasing concentration of the quenching solution will deteriorate its quenching modification effect. For example, when LSCF is quenched in 3.4 mol/L Y^3+^ solution, the thermal expansion coefficient of the modified LSCF is smaller than that of the quenching in the solution of 4.5 mol/L Y^3+^. 

To investigate the effect of the quenching process on the thermal expansion coefficient of commercial materials, a quenching water experiment was conducted. The commercial LSCF material was heated from room temperature to 800 °C in a muffle furnace at 5 °C/min. The LSCF material was quickly removed from the muffle furnace, quenched and cooled in prepared deionized water, and immersed for 3 min. Vacuum filtration was used to collect the treated LSCF material, which was placed in a 60 °C oven for later use.

It is worth noting that commercial materials can also effectively reduce their thermal expansion coefficient through water quenching. Quenching water treatment can also reduce the thermal expansion coefficient, which can be attributed to the fact that the quenching process can increase the oxygen vacancies in materials. 

## 4. Structural Analysis

**Figure 2:**
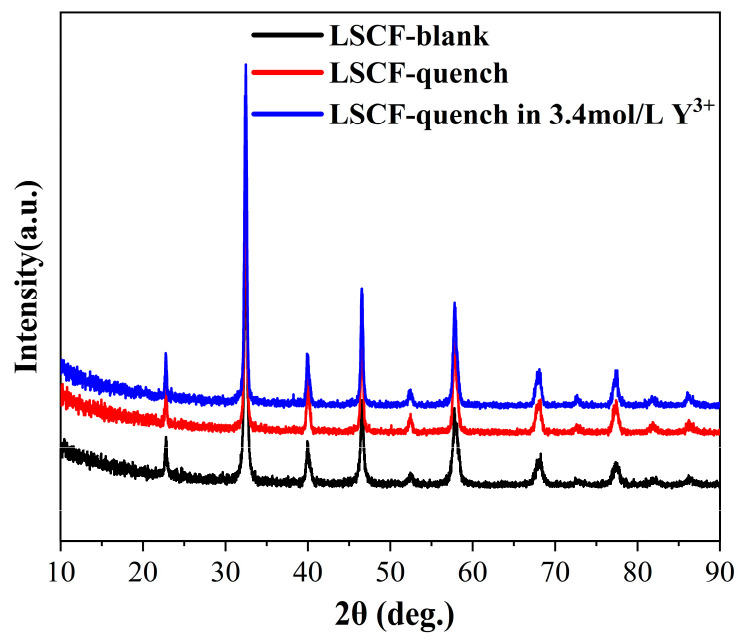
Material XRD characterization.

Using XRD, we analyzed the crystal structure of the quenched materials and compared quenching water (quenching in 4.5 mol/L Y(NO_3_)_3_) and a blank sample. The XRD data are shown in Figure 2. The quenched sample and blank sample highly overlap in the XRD spectrum (Figure 2), indicating that the quenching process does not have any negative impact on the crystal structure of the LSCF material.

## 5. Result Analysis 

**Figure 3:**
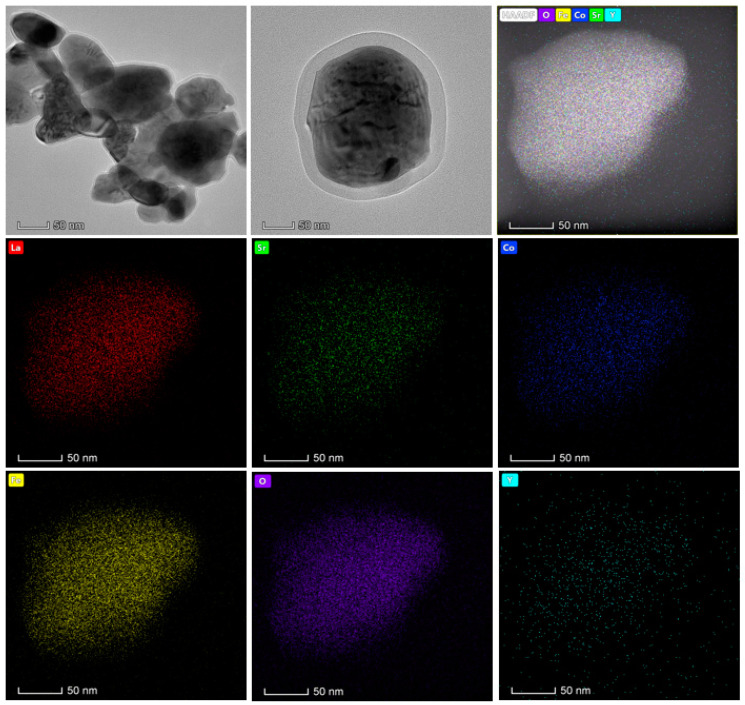
TEM and mapping.

To investigate the reason why quenching in 4.5 mol/L Y(NO_3_)_3_ could significantly reduce the thermal expansion coefficient of the commercial LSCF material, we examined TEM data, which indicated that the quenching process can easily achieve element doping. The mapping element analysis in Figure 3 clearly shows that the material contains elements such as La, Sr, Co, Fe, O, Y, etc. Among them, the Y element shows a small and irregular distribution feature, while La, Sr, Co, Fe, O, and other elements show an aggregated state. The mapping data results show that the Y element was successfully doped into the material after quenching, and the successful doping of the Y element can effectively reduce the material’s thermal expansion coefficient and improve the material’s conductivity. 

At the same time, from the TEM image, it is clearly visible that the commercial material after quenching showed no obvious agglomeration or particle damage, and the application of this process did not have any negative impact on the particle size of the material. 

The quenching process is a simple, low-cost, and universal research strategy to achieve the controllable preparation of the thermal expansion coefficient of the commercial material La_0.6_Sr_0.4_Co_0.2_Fe_0.8_O_3−δ_ (LSCF) through doping elements and increasing oxygen vacancies in the material. The quenching process can directly treat commercial raw materials, improving the optimization process of SOEC powder. In the future, it may be possible to use the quenching process to select appropriate doping elements in order to achieve similar thermal expansion coefficients in SOECs.

## 6. Conclusions

This is the first report on the application of quenching technology in the field of SOECs, providing a simple way to handle materials and easily achieve material defect manufacturing and element doping. This study regulated the type and concentration of quenching salt solution to achieve a controllable preparation of the thermal expansion coefficient of the commercial material LSCF, solving the problem of cell interface delamination caused by mismatched thermal expansion coefficients of commercial materials.

The quenching process meets the development needs of the SOEC industry. It is economical, efficient, and only involves micro-treatment of the material, without negatively affecting the crystal structure and particle size of the material. It does not affect the mixing of back-end slurry or the sintering process of cells. The application and reporting of the quenching process in the field of solid electrolytic cells have certain promoting effects on improving the production process of SOECs.

## Figures and Tables

**Table 1:** Comparing the quenching process with the existing modified material thermal expansion coefficient method.

Serial Number	Improvement Methods	Disadvantages or Advantages	Reference
1	Element doping	Disadvantages: The experimental methods are cumbersome.	[19]
2	Introduction of A-site defects into perovskite	Disadvantages: The experimental methods are cumbersome.	[20]
3	Composite of perovskite electrode materials and electrolyte materials	Disadvantages: The problem of mismatch in material thermal expansion has not been fundamentally solved.	[21]
4	Developing new sintering processes for cells	Disadvantages: Long experimental cycle, high cost, and low success rate.	[22]
5	New material development	Disadvantages: Long experimental cycle, high cost, and low success rate.	[23]
6	Acid-treated electrolyte	Disadvantages: By increasing the contact area of materials, there is no fundamental solution to the problem of the mismatched thermal expansion of materials.	[24]
7	Introducing materials with negative thermal expansion coefficients	Disadvantages: Suitable for special types of materials, unable to modify commercial materials, and the method is not universal.	[11]
8	Quenching process	Advantages: The method is simple, universal, and effectively regulates the thermal expansion coefficient of materials.	This work

## Data Availability

Data are contained within the article.
